# Factors that influence the redox state in children: An exploratory study

**DOI:** 10.6061/clinics/2018/e335

**Published:** 2018-10-10

**Authors:** Sandra Trindade Low, Ana Paula Costa Rodrigues Ferraz, Regiane Maio, Fabiane Valentini Francisqueti, Damiana Tortolero Pierine, Weidylla Natália Silva Borges, Ariel Dandara de Carvalho, Gedalva Pereira de Lima, Ana Lúcia A Ferreira, Klinsmann Carolo dos Santos, Camila Renata Corrêa

**Affiliations:** I Universidade de Pernambuco (UPE), Recife, PE, BR; II Universidade Federal de Pernambuco, Recife, PE, BR; III Universidade Estadual Paulista (UNESP), Faculdade de Medicina, Botucatu, SP, BR

**Keywords:** Oxidative Stress, Breastfeeding, Antioxidant Imbalance

## Abstract

**OBJECTIVE:**

The aim of the present study was to investigate the association of the redox state via malondialdehyde (MDA) as a lipid peroxidation biomarker and hydrophilic antioxidant capacity (HAC) with dietary, anthropometric, demographic, socio-economic and clinical variables as well as the serum concentrations of vitamins in children aged 20-36 months. This cross-sectional study was conducted from May 2013 to May 2014 and included a total of 100 children.

**METHODS:**

The variables studied included anthropometric measurements, dietary intake by the Food Frequency Questionnaire (FFQ), socio-demographic features, clinical attributes, serum redox status, and serum vitamin concentrations.

**RESULTS:**

Children with a family income above the minimum wage and adequate body mass index (BMI) presented higher HAC. The MDA concentration was higher in children older than 24 months. Breastfeeding for up to 120 days provided greater antioxidant capacity. Children classified in the 2^nd^ tertile for “fruit and vegetables” and “milk and dairy products” consumption showed lower levels of MDA. There was a positive correlation of MDA with serum vitamin A levels. These results show that among children in the 20-36 months age group, family income, breastfeeding, BMI and intake of fruits and vegetables can have an influence on the imbalance of the redox state.

**CONCLUSION:**

One strategy to prevent the imbalance between oxidants and antioxidants could be for health professionals to raise awareness among families, as such knowledge could repress/prevent the progression/initiation of several diseases in adult life.

## INTRODUCTION

Good feeding habits in the first years of life and early childhood are very important. During this phase of life, the consumption of nutrient-rich foods is necessary for adequate growth and development, intestinal maturation, strengthening of the immune system, and formation of antioxidant defenses 1. The risk of overweight and obesity in childhood can be influenced by feeding practices such as breastfeeding, the introduction of complementary foods 2 and nutritional deficiencies that may trigger metabolic programming pathways, thereby predisposing children to chronic diseases in later life 3. Furthermore, some studies suggest that exposure to different foods in early life can potentially predict food preferences, and eating behaviors have been shown to persist across the life course 4. Together, these factors show the relevance of protecting future health through good nutrition practices in early life.

Poor eating habits provide fewer exogenous antioxidants and lead to excessive macronutrient oxidation that could affect endogenous antioxidant defenses 5. Moreover, this shift in eating behavior may result in changes in body composition, leading to obesity, overweight, and/or malnutrition 6. It is also known that some determinants influence the health of children, such as early weaning and a low intake of vitamins and antioxidant sources 7. All of these factors are directly related to the alteration of the redox state and oxidative stress of young individuals 8.

The imbalance between oxidant and antioxidant substances is defined as oxidative stress 9. Both substances (oxidants and antioxidants) are produced via oxidation-reduction reactions, and because the generation and the activity of oxidants and antioxidants depend on these reactions, authors have used the definition of redox system imbalance to refer to the oxidative stress condition 10–12. Studies have shown that excessive production of reactive oxygen species (ROS) and nitrogen species (RNS) is involved in various diseases through lipid peroxidation and oxidation of sugars and proteins, leading to dysfunction and possibly cell death 9. Therefore, the measurement of redox status and nutritional monitoring in early life are important in order to prevent complications caused by oxidative stress-related disorders both in childhood and adulthood 13.

In this context, the aim of this study was to investigate the association of the redox state via the biomarker of lipid peroxidation (malondialdehyde; MDA) and hydrophilic antioxidant capacity (HAC) with dietary behavior, anthropometric measurements, demographic, socioeconomic and clinical variables, and the serum concentrations of vitamins in children between 20 and 36 months of age who attended the childcare service of the Integrated Health Center Amaury de Medeiros (CISAM), University of Pernambuco, Recife – PE, northeastern Brazil.

## MATERIALS AND METHODS

### Study protocol

A cross-sectional study was conducted from May 2013 to May 2014 at CISAM of the University of Pernambuco, Recife – PE/Brazil. Two hundred fifty-eight (258) children aged 20-36 months were selected based on the medical records of the institution. Of these, one did not agree to participate in the study; it was not possible to make telephone contact with 146, and 11 were excluded due to chronic and infectious diseases. Therefore, a total of 100 children were included in the study (recruited). Participation in the study was voluntary after obtaining consent from the legal representatives, under the rules of Resolution 466/12, which addresses ethics in research with human subjects. We performed the study according to the guidelines established by the Declaration of Helsinki, and this study was approved by the Research Ethics Committee of CISAM (CEP-CISAM) - CAAE 14518113.2.0000.5191.

### Nutritional and social variables

The nutritional variables studied included the following anthropometric measures: weight / age (W / A), height / age (H / A), body mass index / age (BMI / A), and dietary intake. Anthropometric evaluation considered the measurements obtained from the Child Health Booklet (2011) World Health Organization 14. The anthropometric variables were expressed as a Z score. Dietary intake was assessed by the Food Frequency Questionnaire (FFQ), a qualitative retrospective assessment of the previous 6 months, adapted from the semi-quantitative FFQ for children 2 to 5 years of age proposed by Colucci et al. 15. Dietary foods were grouped in five categories: 1) carbohydrates, 2) meat and eggs, 3) milk and dairy products, 4) fruit and vegetables, and 5) processed foods. The FFQ included 7 rate of consumption categories: not less than 1 time / month, 1 to 3 times / month, 1 time / week, 2 to 4 times / week, one time per day, and 2 times or more / day. To change the frequency of the individual consumption score, it was calculated by the formula proposed by Fornés et al. 16. This score ranged from 0 (zero) for food ever eaten to 1 (one) for food consumed two or more times per day.

All the data regarding the children were acquired in an interview with the mothers based on the applied questionnaires and from the medical records.

### Evaluation of redox state

As described previously 17, 5 mL of venous blood was collected for the analysis of MDA, HAC, retinol, β-carotene and α-tocopherol. The blood was centrifuged for 10 min at 3,000 rpm at 4°C, and the serum was aliquoted and stored at -80°C until the time of analysis. The level of MDA (mol/L) was determined by high-performance liquid chromatography (HPLC) with a fluorescence detector (Waters 2475, Waters Alliance, Milford, Massachusetts, USA), which was calibrated with excitation at 515 nm and emission at 553 nm. The mobile phase consisted of 20 mM potassium phosphate and acetonitrile (80:20, v / v) with a flow of 0.8 mL / min 18. HAC was determined fluorometrically, as described by Beretta et al. 12, by comparing the area under the curve relating to the oxidation kinetics of the suspension phosphatidylcholine (PC), which was used as a reference biological matrix, using a Victor X2 reader (PerkinElmer -Boston, MA). The retinol, α-tocopherol and β-carotene levels were measured by HPLC in a C_30_ column (Waters carotenoid -YMC: 4.6 mm x 150 mm; 3.0 μm). As described by Correa et al. 17, the plasma samples (200 µL) were extracted with 2 mL of chloroform:methanol (2:1) followed by 3 mL of hexane. Samples were dried under nitrogen air and resuspended in 75 µL ethanol:methyl tert-butyl ether (2:1), of which 25 µL was injected into the HPLC. The HPLC system consisted of a Waters 2695 Separation Module, a 2996 Photodiode Array Detector, a Waters 2475 Multi λ Fluorescence Detector, a C_30_ carotenoid column (3 µm, 150 mm × 3.0 mm, YMC, Wilmington, NC), and a Waters Millenium 32 data station. The mobile phase was methanol:methyl tert-butyl ether:water (85:12:3 by volume with 1.5% ammonium acetate in water; solvent A) and methanol:methyl tert-butyl ether:water (8:90:2 by volume with 1% ammonium acetate in water; solvent B). The results were adjusted using an internal standard of echinenone. The inter-assay (n=25) and intra-assay (n=9) coefficients of variation (CV) were both 4%. Recovery of the internal standard averaged 97%.

### Statistical analysis

For comparison of the categories of independent variables in relation to oxidative stress variables, the data were analyzed with Student's t-tests with equal variances and unequal variance or the Mann-Whitney test for comparing two categories. Multiple comparisons were made with the F test (ANOVA) followed by the Tamhane test or the Kruskal-Wallis test for comparisons of more than two categories. The association between categorical variables was evaluated by the chi-square test or Fisher's exact test when the condition for using the chi-square test was not observed. Student's t-tests and the F tests (ANOVA) were applied for checking the normality assumption, and the Mann-Whitney and Kruskal-Wallis tests were used in cases of rejection of the hypothesis of normality. The verification of the equality of variances was performed by Levene's F test. The relationship between food intake and oxidative stress was distributed into tertiles: 1^st^ tertile - 0 to 33.3; 2^nd^ tertile - 33.3 to 66.7; and 3^rd^ tertile >66.7. The margin of error used in the statistical tests was 5%. Data were entered in an Excel spreadsheet, and the program used for statistical calculations was SPSS (Statistical Package for Social Sciences) version 21.

## RESULTS

Among the 100 children, 59% were aged between 20 and 24 months, and 54% were male. Sixty percent of the families received up to one minimum wage (family income), and the mother's education average was 10 years. Regarding the number of queries in the Child Care Clinic, 56% had nine or more queries in this service. It was determined that 86% of subjects were not taking any medication, and 75% never had a serious illness that required hospitalization. The anthropometric indices of weight / age and height / age were adequate in 92% and 94% of children, respectively. In relation to BMI, 66% of the sample was classified as normal weight, 16% at risk of overweight, 16% obese and 2% undernourished. Table 1 shows the association of redox state and social and nutritional variables. Those with a family income above the minimum wage category presented higher HAC than children from families with a lower family income. The same was observed for the association between BMI and HAC, in which children with appropriate BMI had higher antioxidant protection (HAC). The age of the children was also associated with the concentration of MDA; children under 24 months had higher levels of lipid peroxidation than older children. There was no association between the variables of the redox state with the mother's education, gender and number of queries in the childcare service.

Breastfeeding for up to 120 days provided greater antioxidant capacity than a period lasting 31 to 120 days, but there was no difference for serum MDA levels (Table 2).

Supplementation with vitamins and medication use were not associated with the redox state of these children. In the nutritional assessment, according to the mean scores shown in Table 3, it was determined that the most consumed food group was milk and milk products (0.41±0.17), followed by carbohydrates (0.33±0.11), meat and eggs (0.26±0.17), fruits and vegetables (0.26±0.15), and industrial products (0.18±0.12). The association between the consumption of these food groups and the redox state showed that children classified in the 2^nd^ tertile for “fruit and vegetables” and “milk and dairy products” consumption had lower levels of MDA. There was no difference in the redox state in relation to the consumption of carbohydrates, meat and eggs and processed foods.

There was a negative correlation between weight and BMI with HAC. A positive correlation was found for MDA with vitamin A serum levels, but no correlation was found between the other variables, as shown in Table 4.

## DISCUSSION

The present study was conducted to investigate the association of the redox state via the lipid peroxidation biomarker (MDA) and hydrophilic antioxidant capacity (HAC) with dietary consumption, anthropometric, demographic, socioeconomic and clinical variables, and serum concentrations of vitamins in children between 20 and 36 months of age who attended the childcare service of the Integrated Health Center Amaury Medeiros (CISAM) Recife (PE) in northeastern Brazil.

Among the social and nutritional variables examined (Table 1), the present study showed that antioxidant capacity (HAC) was higher in children with a higher family income. This fact may be related to the ability to purchase healthier foods, which tend to be more expensive 19. Additionally, in the first years of life, the newborn's health could be influenced by the maternal perception of the child's nutritional status, as the mother's education may contribute to misperceptions about underestimating or overestimating her child's height and weight 20.

Additionally, children with an appropriate BMI showed a higher level of antioxidant protection. These results are in agreement with the literature, indicating that nutritional disorders such as obesity, overweight and / or malnutrition are conditions in which there is an imbalance in the redox system 21. These findings were confirmed when performing the correlation of HAC and MDA with BMI, weight and height (Table 4). There was a negative correlation between weight and BMI parameters relative to HAC. Briefly, the overweight- and obesity-related nutritional disorders presented in this study decreased the serum antioxidant capacity. These findings are in agreement with those of Vehapoglu et al. 22, who assessed children aged two to eleven years and found that obesity decreased the antioxidant capacity. Overweight and obesity are defined as an excessive accumulation of fat mass in cells known as adipocytes, and their occurrence can be explained by adipocyte hypertrophy, hyperplasia, or a combination of both 23. The state of high fat mass accumulation is favorable as an oxidizing environment through the differentiation of preadipocytes and maintenance of the metabolic function of adipocytes, triggering an imbalance of the redox system 24. Additionally, when lipids such as polyunsaturated fatty acids are oxidized, they create a product of lipoperoxidation, known as MDA 25, which is associated with body weight alterations 26. In our study, MDA levels were higher in children below 24 months of age. The literature shows that, in this phase of life, the balance between the formation and removal of reactive oxygen species may be unstable because the antioxidant defense is still immature 27,28, which may be reflected in the MDA levels.

The role of oxidative stress in diseases such as retinopathy of prematurity, necrotizing enterocolitis and prenatal brain damage in the newborn period share mechanisms in common with the pathology of ischemia-reperfusion injury through the generation of ROS during the metabolism of ATP. The product of ATP degradation (hypoxanthine) is metabolized by the enzyme xanthine dehydrogenase to xanthine oxidase under disease conditions, which releases ROS as superoxide anions 29. The physiopathology of diseases that involve inflammatory processes and ROS production contributes to lipid peroxidation and low levels of endogenous antioxidant enzymes and vitamins A and E 30.

Breastfeeding should be the primary source of food offered to newborns. In the second year of life, breast milk remains an important source of nutrients, which may help the development, immunity, and maintenance of a balanced redox system 31. It is estimated that two cups (500 mL) of milk in the second year of life provide 95% of vitamin C requirements, 45% of vitamin A, 38% of protein and 31% of the total energy needed for children. In addition, milk continues to protect against infectious diseases. Studies on three continents found that when children were not breastfed in the second year, their risk of dying from infectious disease was almost twice that of breastfed infants 14. Thus, breast milk contributes to health as a source of endogenous and exogenous molecules from vitamins (A, E, and C), minerals (Cu, Zn, and Se), endogenous enzymes (SOD and CAT), iodine 32, bioactive factors such as immunoglobulins (IgA/sIgA, IgG, and IgM), cytokines (IL-6, IL-7, and IFN- γ), growth factors (EGF and BNDF), hormones and metabolic factors (calcitonin, lactoferrin, adiponectin, and leptin). Together, these factors recruit immunological cells, protect against infections, enhance intestinal and neuronal development and stimulate the antiviral and anti-inflammatory systems 33,34. In fact, the molecules contributing to the total antioxidant status in newborns can act in a synergistic manner to neutralize free radicals 32.

Vitamin A deficiency in some regions of Brazil is still a problem, with studies showing that more than 10% of children have plasma levels below 20 μg / dL (>0.70 μmol / L), which, according to the World Health Organization, characterizes vitamin A deficiency 35. For this reason, vitamin supplementation is a practice adopted by health professionals 2.

Our data show that children who are breastfed for more than 120 days showed higher antioxidant capacity when compared to those who received breast milk for a shorter period (Table 2). In addition, the use of multivitamins and vitamin A was not associated with indicators of the redox system. However, there was a positive correlation between the plasma levels of vitamins A and MDA. There was also an association between the intake of fruits and vegetables and MDA in children classified in the 3^rd^ tertile (Table 3), as higher consumption of this food source was associated with higher MDA values when compared to other tertiles. It is assumed that this association may be related to the excessive intake of vitamin A from various food sources (feeding and introduced in fortified foods) 28.

The main mechanism for the correlation between vitamin A and MDA is the oxidative stress of lipid metabolism caused by prooxidant effects. Stress begins from changes in cellular homeostasis, creating intermediate products of the peroxidation of polyunsaturated fatty acids, which in turn leads to the formation of the lipoperoxyl radical (LOO•) and highly reactive molecules such as ROS. Due to their molecular susceptibility for instability, these products create new aldehyde derivatives such as MDA 36.

To maintain the redox balance of free radical formation and function, antioxidant networks are required, acting by adding an oxygen or removing a hydrogen or electron from free radicals. In particular, vitamin A is a carotenoid produced by β-carotene breakdown, which has the ability to neutralize peroxyl radicals formed from lipid peroxidation or physiological maintenance 37. However, excessive intake by feeding or the consumption of fortified foods, as shown in our study, promoted an excess carotenoid concentration in cell membranes, which may increase the membrane permeabilization for ROS aqueous and autoxidation 38, promoting oxidative stress and lipid peroxidation and raising MDA levels.

Additionally, the association between the intake of fruits and vegetables and MDA in children classified in the 3^rd^ tertile (Table 3) can be explained by the retinol variable, through the higher consumption of this food source. Most reports in the literature show that diets rich in vegetables and fruits are good sources of antioxidants, especially those that our body is not able to produce, such as vitamin E, vitamin C, phenolic compounds, carotenoids and trace metals (selenium, manganese, zinc), thereby preventing many diseases and acting as antioxidants 39. Phenolic compounds are the most abundant components in fruits and vegetables, and their prooxidant effect is related to vitamin C combined with minerals such as iron and copper, which reduces Fe^3+^ to Fe^2+^ and reduces H_2_O_2_ to –OH radicals 37; additionally, the oxidation of the enzymatic endogenous defense in a biological system contributes to redox imbalance 40. Our results show that family income, breastfeeding, BMI, and consumption of fruits and vegetables influence the balance of the redox state in children between 20 and 36 months. One strategy to prevent the imbalance between oxidants and antioxidants, which is a delicate system in this period of life, could be for health professionals to raise awareness among families, which could prevent the progression/initiation of several diseases in adult life.

## AUTHOR CONTRIBUTIONS

All authors contributed to the study design, interpretation, analysis and review of the final version of the manuscript. Low ST, Maio R, Borges WN, Carvalho AD and Lima GP recruited the participants, conducted the interviews and analyzed the data. Pierine DT contributed to data analysis and data interpretation. Francisqueti FV and Ferreira AL participated in the experimental design and interpretation of the data. dos Santos KC, Ferraz APCR and Correa CR analyzed, revised and wrote the manuscript.

## Figures and Tables

**Table 1:** Association between HAC, MDA and social and nutritional variables.

Variable	HAC (%)	MDA (µmol/L)
	Mean±SD (Median)	Mean±SD (Median)
Mothers' education (years)		
<9	40.63±10.38 (38.58)	1.30±0.72 (0.98)
≥9	41.73±11.61 (41.92)	1.24±0.63 (1.16)
*p* value	*p*(1)=0.436	*p*(1)=0.860
Family income		
<1	39.65±10.45 (39.93)	1.30±0.66 (1.22)
≥1	44.20±11.99 (43.26)	1.17±0.63 (1.10)
*p* value	*p*(2)=0.048*	*p*(1)=0.329
Children’s ages (month)		
<24	42.72±11.83 (42.25)	1.38±0.66 (1.37)
≥24	39.56±10.17 (39.93)	1.07±0.60 (0.92)
*p* value	*p*(2)=0.181	*p*(1)=0.015*
Gender		
Female	41.83±11.69 (41.52)	1.26±0.67 (1.22)
Male	41.07±10.93 (41.70)	1.25±0.64 (1.02)
*p* value	*p*(1)=0.893	*p*(1)=0.967
BMI		
Nutrition disorder	37.30±10.74 (39.73)	1.40±0.71 (1.35)
Adequate	43.55±10.97 (42.47)	1.18±0.61 (1.00)
*p* value	*p*(1)=0.023*	*p*(1)=0.145
Number of doctor visits to monitor		
<9	39.42±10.79 (39.48)	1.31±0.68 (1.10)
≥9	42.99±11.43 (42.27)	1.21±0.63 (1.14)
*p* value	*p*(2)=0.116	*p*(1)=0.518

(*): *p*.

(1): Mann-Whitney test.

(2): Student’s t-test with equal variance.

**Table 2:** Association between HAC, MDA and clinical variables.

Variable	HAC (%)	MDA(µmol/L)
	Mean±SD (Median)	Mean±SD (Median)
Breastfeeding (days)		
<120	37.45±10.16 (39.68) (A)	1.30±0.73 (0.95)
>120	45.39±11.12 (44.37) (B)	1.17±0.58 (1.10)
*p* value	*p*(1)=0.014*	*p*(1)=0.724
Use of multivitamins		
Yes	39.86±9.64 (42.35)	1.29±0.58 (1.22)
No	41.81±11.62 (41.30)	1.24±0.67 (1.09)
*p* value	*p*(2)=0.663	*p*(2)=0.544
Vitamin A intake		
Yes	41.82±11.33 (42.04)	1.27±0.66 (1.14)
No	35.15±7.89 (38.18)	0.99±0.33 (0.99)
*p* value	*p*(2)=0.074	*p*(2)=0.392
Use of drugs		
Yes	41.54±4.72 (40.40)	1.39±0.56 (1.42)
No	41.40±11.99 (41.62)	1.23±0.66 (0.96)
*p* value	*p*(5)=0.939	*p*(2)=0.233

(*): *p*.

(1): Kruskal Wallis test.

(2): Mann-Whitney test.

(3): Test F (ANOVA).

(4): Student’s t-test with equal variance.

(5): Student’s t-test with unequal variances.

**Table 3:** Association between HAC, MDA and the food groups consumed by tertiles.

Variables	HAC (%)	MDA (µmol/L)
	Mean±SD (Median)	Mean±DP (Median)
Carbohydrate		
1^st^ Tertile (<0.28)	38.44±10.40 (40.02)	1.20±0.65 (1.10)
2^nd^ Tertile (0.28 to 0.37)	43.23±12.60 (41.14)	1.25±0.61 (1.22)
3^rd^ Tertile (>0.37)	41.74±9.43 (42.53)	1.31±0.72 (1.00)
*p* value	*p*(1)=0.319	*p*(1)=0.804
Fruit and vegetables		
1^st^ Tertile (<0.17)	39.55±10.31 (40.17)	1.24±0.67 (0.96) (AB)
2^nd^ Tertile (0.17 to 0.32)	42.21±12.41 (40.46)	1.06±0.52 (0.93) (A)
3^rd^ Tertile (>0.32)	42.50±10.85 (42.78)	1.49±0.71 (1.44) (B)
*p* value	*p*(2)=0.507	*p*(1)=0.048*
Meat and eggs		
1^st^ Tertile (<0.17)	42.64±11.61 (43.01)	1.13±0.71 (0.90)
2^nd^ Tertile (0.17 to 0.31)	40.95±12.48 (40.13)	1.25±0.58 (1.18)
3^rd^ Tertile (>0.31)	40.79±9.47 (41.52)	1.38±0.66 (1.10)
*p* value	*p*(1)=0.538	*p*(1)=0.153
Milk and derivatives		
1^st^ Tertile (<0.33)	42.63±9.73 (41.90)	1.43±0.68 (1.39) (A)
2^nd^ Tertile (0.33 to 0.43)	41.17±13.99 (42.40)	1.04±0.64 (0.82) (B)
3^rd^ Tertile (>0.43)	40.40±8.98 (40.10)	1.33±0.57 (1.40) (A)
*p* value	*p*(2)=0.727	*p*(1)=0.009*
Industrialized		
1^st^ Tertile (<0.12)	42.22±8.90 (42.54)	1.23±0.73 (1.11)
2^nd^ Tertile (0.12 to 0.21)	40.71±13.37 (40.78)	1.17±0.57 (1.05)
3^rd^ Tertile (>0.21)	41.80±9.68 (41.52)	1.41±0.69 (1.30)
*p* value	*p*(2)=0.842	*p*(1)=0.358

(*): *p*.

(1): Kruskal Wallis test.

(2): F Test (ANOVA).

Different letters indicate significant differences between the categories.

**Table 4:** Correlations between HAC and MDA with numeric variables.

Variables	HAC (%)	MDA (µmol/l)
	r (p)	r (p)
Anthropometric data		
Weight	-0.199 (0.047)*	0.012 (0.908)
Height	0.001 (0.991)	-0.105 (0.298)
BMI	-0.315 (0.001)*	0.126 (0.210)
Plasmatic levels		
Vitamin A	0.026 (0.796)	0.230 (0.021)*
Vitamin E	-0.142 (0.158)	-0.087 (0.391)
Beta-carotene	-0.036 (0.719)	-0.050 (0.623)
Food group scores		
Carbohydrate	0.065 (0.518)	0.051 (0.614)
Fruits and vegetables	0.085 (0.399)	0.135 (0.180)
Meet and eggs	-0.086 (0.394)	0.149 (0.138)
Milk and derivatives	-0.055 (0.587)	-0.043 (0.668)
Industrialized	0.002 (0.987)	0.108 (0.284)

Spearman and Pearson tests (*): *p*<0.05.

HAC, hydrophilic antioxidant capacity; MDA, malondialdehyde.
